# Deletion of the Murine Ortholog of the Human 9p21.3 Locus Leads to Insulin Resistance and Obesity in Hypercholesterolemic Mice

**DOI:** 10.3390/cells13110983

**Published:** 2024-06-05

**Authors:** Sanna Kettunen, Tuisku Suoranta, Sadegh Beikverdi, Minja Heikkilä, Anna Slita, Iida Räty, Elias Ylä-Herttuala, Katariina Öörni, Anna-Kaisa Ruotsalainen, Seppo Ylä-Herttuala

**Affiliations:** 1 A.I. Virtanen Institute, University of Eastern Finland, 70210 Kuopio, Finland; sanna.kettunen@uef.fi (S.K.); tuisku.suoranta@uef.fi (T.S.); sadegh.beikverdi@helsinki.fi (S.B.); minja.heikkila@uef.fi (M.H.); anna.slita@uef.fi (A.S.); iida.raty@uef.fi (I.R.); elias.yla-herttuala@uef.fi (E.Y.-H.); seppo.ylaherttuala@uef.fi (S.Y.-H.); 2 Imaging Center, Kuopio University Hospital, 70200 Kuopio, Finland; 3 Wihuri Research Institute, 00290 Helsinki, Finland; kati.oorni@wri.fi

**Keywords:** lncRNA, 9p21.3, ANRIL, Ak148321, insulin resistance, obesity, Cdkn2a/b, Chr4^Δ70/Δ70^

## Abstract

The 9p21.3 genomic locus is a hot spot for disease-associated single-nucleotide polymorphisms (SNPs), and its strongest associations are with coronary artery disease (CAD). The disease-associated SNPs are located within the sequence of a long noncoding RNA ANRIL, which potentially contributes to atherogenesis by regulating vascular cell stress and proliferation, but also affects pancreatic β-cell proliferation. Altered expression of a neighboring gene, *CDKN2B*, has been also recognized to correlate with obesity and hepatic steatosis in people carrying the risk SNPs. In the present study, we investigated the impact of 9p21.3 on obesity accompanied by hyperlipidemia in mice carrying a deletion of the murine ortholog for the 9p21.3 (Chr4^Δ70/Δ70^) risk locus in hyperlipidemic *Ldlr*^−/−^*ApoB*^100/100^ background. The Chr4^Δ70/Δ70^ mice showed decreased mRNA expression of insulin receptors in white adipose tissue already at a young age, which developed into insulin resistance and obesity by aging. In addition, the *Sirt1-Ppargc1a-Ucp2* pathway was downregulated together with the expression of *Cdkn2b,* specifically in the white adipose tissue in Chr4^Δ70/Δ70^ mice. These results suggest that the 9p21.3 locus, ANRIL lncRNA, and their murine orthologues may regulate the key energy metabolism pathways in a white adipose tissue-specific manner in the presence of hypercholesterolemia, thus contributing to the pathogenesis of metabolic syndrome.

## 1. Introduction

As the levels of obesity are increasing on a global level, its effects on human health due to dysregulated lipid, glucose, and insulin metabolism, together chronic low-grade inflammation and oxidative stress, are becoming more and more significant [1,2,3]. Hypercholesterolemia is a well-recognized risk factor for cardiovascular diseases (CVDs), as it promotes lipid accumulation in the arterial wall and enhances cholesterol and lipid loading into adipocytes [4,5]. The 9p21.3 genomic locus has been identified as a hot spot for many disease-associated single-nucleotide polymorphisms (SNPs). The strongest associations have been reported with coronary artery disease (CAD), but some SNPs in the region have also been linked to other diseases, such as type 2 diabetes (T2D) and several cancers [6,7,8,9]. Despite the common risk factors of CVDs and T2D, the 9p21.3 SNPs associated with CAD have been reported to be independent and not associated with the risk of T2D [10].

The locus harboring the CAD risk SNPs does not contain any protein-coding genes, but instead, it contains the sequence for *CDKN2B-AS1*, also known as an Antisense Noncoding RNA in the INK4 Locus (ANRIL) [10,11]. Two cyclin-dependent kinase inhibitors (*CDKN2A* and -*B*) and methylthioadenosine phosphorylase (*MTAP*) neighbor the ANRIL sequence, and there is evidence to suggest that ANRIL contributes to their regulation in a tissue-specific manner [11,12,13,14].

Initially, the CAD risk associated with 9p21.3 genetic variants and ANRIL expression was reported being independent of traditional CAD risk factors like sex, smoking, obesity, hypertension, or diabetes [15,16,17]. However, recent studies have reported 9p21.3 risk variants having an effect on plasma lipid profile and lipid uptake in vascular cells [18,19]. The VITA cohort study also revealed that elevated circulating levels of ANRIL may predict later onset of T2D [20]. In addition, a T2D risk SNP in ANRIL exon 2 was reported to associate with elevated ratios of circular to linear isoforms of ANRIL and was found to reduce pancreatic β-cell proliferation [21]. The genes neighboring ANRIL have also been reported to have a role in metabolic regulation, as *CDKN2B* is highly expressed in subcutaneous adipose tissue (SAT), with the levels positively correlating with obesity and hepatic steatosis in 9p21.3 risk allele carriers [22]. The study proposed that *CDKN2B* regulates adipocyte proliferation and affects postprandial triacylglycerol clearance by inhibiting SAT fatty acid trafficking. Also, the deletion of *Cdkn2a* in a mouse model protected against diet-induced obesity, possibly by regulating adipocyte browning [23].

A mouse model with a deletion located to the ortholog of the human 9p21.3 risk interval (Chr4^Δ70/Δ70^) has been shown to exhibit an increased body weight and tumor occurrence in wild-type background, as well as more advanced atherosclerosis in hyperlipidemic mice on a high-fat diet (HFD) [24,25,26]. Moreover, myeloid-specific Chr4^Δ70/Δ70^ deletion also elevated the body weight and plasma lipid and fasting glucose levels in hyperlipidemic mice after a prolonged HFD [26]. Similarly to humans, the mouse locus also encodes a lncRNA, and like the human ANRIL, the murine lncRNA Ak148321 produces both linear and circular isoforms.

In the present study, we aimed to investigate the effects of the 9p21.3 CAD risk locus on obesity in response to hypercholesterolemia by characterizing the metabolic phenotype of mice carrying the deletion of murine 9p21.3 ortholog (Chr4^Δ70/Δ70^) in the hyperlipidemic *Ldlr*^−/−^*ApoB*^100/100^ background. Here, we show that Chr4^Δ70/Δ70^ leads to impaired expression of insulin receptors and *Cdkn2b* in a white adipose tissue-specific manner in the presence of hypercholesterolemia. With aging, this seems to lead to insulin resistance and obesity due to the downregulation of the *Sirt1-Pgc1a-Ucp2* axis. Thus, the deletion of the murine orthologue of the 9p21.3 CAD risk locus may affect the regulation of key energy metabolism pathways in white adipose tissue in the presence of hypercholesterolemia and to contribute to the pathogenesis of metabolic syndrome.

## 2. Materials and Methods

### 2.1. Mice

**Figure 1:**
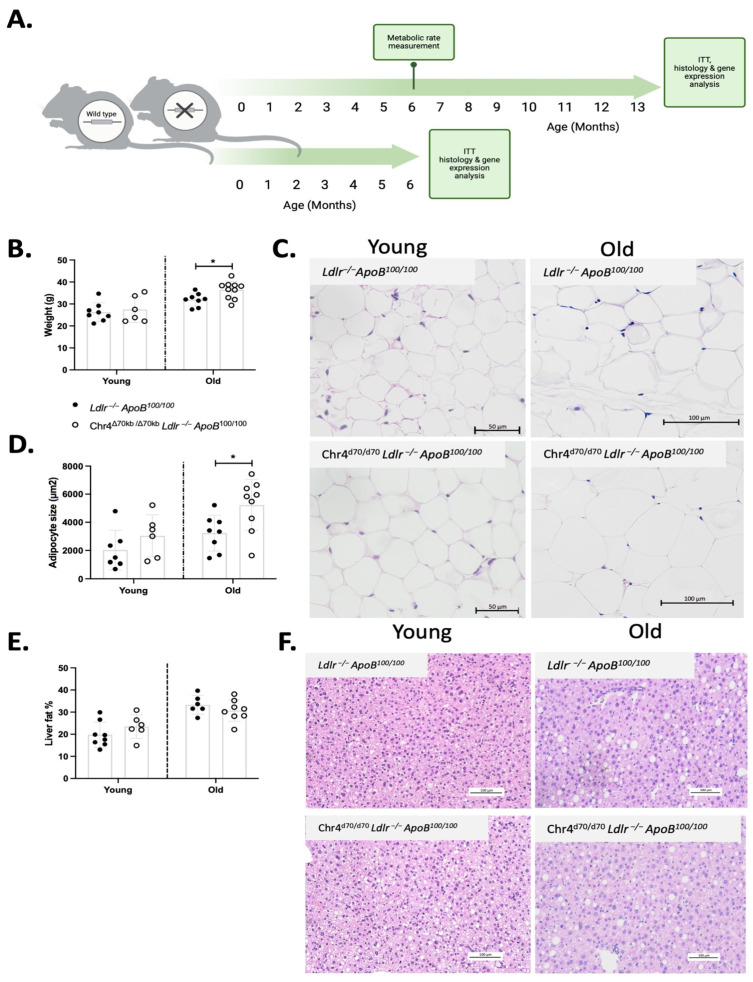
*Chr4^Δ70/Δ70^* in aged *Ldlr^−/−^ApoB^100/100^* mice led to obesity and enlargement of adipocytes. (**A**) Timeline demonstrating the experiments performed in Chr4^Δ70/Δ70^*Ldlr*^−/−^*ApoB*^100/100^ (*n* = 6) and their *Ldlr*^−/−^*ApoB*^100/100^ littermates (*n* = 8) at the age of 6 months and at the age of one year on a standard laboratory diet (Chr4^Δ70/Δ70^ n = 10; *Ldlr*^−/−^*ApoB*^100/100^ *n* = 8). (**B**) Body weight of young Chr4^Δ70/Δ70^ (*n* = 6) and *Ldlr*^−/−^*ApoB*^100/100^ (*n* = 7) and aged Chr4^Δ70/Δ70^ (*n* = 10) and *Ldlr*^−/−^*ApoB*^100/100^ (*n* = 8) mice. (**C**) Representative figure of adipocytes in young and aged Chr4^Δ70/Δ70^ and *Ldlr*^−/−^*ApoB*^100/100^ mice. (**D**) Average adipocyte size of young Chr4^Δ70/Δ70^ (*n* = 6) and *Ldlr*^−/−^*ApoB*^100/100^ (*n* = 7) and aged Chr4^Δ70/Δ70^ (*n* = 9) and *Ldlr*^−/−^*ApoB*^100/100^ (*n* = 8) mice. (**E**) Hepatic steatosis in young Chr4^Δ70/Δ70^ (*n* = 6) and *Ldlr*^−/−^*ApoB*^100/100^ (*n* = 8) and old Chr4^Δ70/Δ70^ (*n* = 8) and *Ldlr*^−/−^*ApoB*^100/100^ (*n* = 6) mice. (**F**) Representative figure of the liver histology in young and aged Chr4^Δ70/Δ70^ and *Ldlr*^−/−^*ApoB*^100/100^ mice. Comparison of *Ldlr*^−/−^*ApoB^1^*^00/100^ and Chr4^Δ70/Δ70^ groups was performed separately for each age group by using *t*-test. Asterisk (*) indicates statistical significance. Differences were considered statistically significant when *p* or adjusted *p* < 0.05.

A Chr4^Δ70/Δ70^ mouse model deficient in the orthologous region to a human 9p21.3 CAD risk interval was backcrossed it into a hyperlipidemic *Ldlr*^−/−^*ApoB*^100/100^ strain, to obtain a human-like lipoprotein profile with a high LDL fraction [24,27]. Chr4^Δ70/Δ70^*Ldlr*^−/−^*ApoB*^100/100^ female mice (*n* = 10) (hereafter referred to as Chr4^Δ70/Δ70^) and their *Ldlr*^−/−^*ApoB*^100/100^ littermate controls (*n* = 8) were aged until one year of age on a standard laboratory diet (Teklad Global 16% Protein Rodent Diet, Inotiv, West Lafayette, IN, USA) (Figure 1A). Halfway through the follow up period, the basal metabolic rate of the mice was measured. Finally, the mice were sacrificed with CO_2_, and the tissues were collected for histopathology and gene expression analyses. In addition, in accordance with the principles of the 3Rs in animal research, we also utilized previously collected tissue samples and unpublished data of young (6 months old) Chr4^Δ70/Δ70^ (*n* = 6) and *Ldlr*^−/−^*ApoB*^100/100^ (*n* = 8) mice that were characterized for atherosclerosis in a previous study [26]. We also performed an additional stress test where we fed aged Chr4^Δ70/Δ70^ (*n* = 7) and *Ldlr*^−/−^*ApoB*^100/100^ (*n* = 8) with a high-fat diet (HFD) for 3 months and recorded the bodyweight and analyzed the insulin response of the mice, as well as characterized the histopathology and gene expression of their white adipose tissue.

All animal work was performed in the Animal Centre of University of Eastern Finland. Housing conditions were controlled for temperature and humidity, the 12 h light/dark cycle was used, and the animals received food and water ad libitum. Animal work was approved by the National Experimental Animal Board of Finland and carried out following the guidelines of the Finnish Act on Animal Experimentation and Directive 2010/63/EU of the European Parliament.

### 2.2. Insulin Tolerance Test, Glucose Tolerance Test, and Insulin Secretion Test

An insulin tolerance test (ITT) was performed using randomly selected young Chr4^Δ70/Δ70^ (*n* = 7) and *Ldlr*^−/−^*ApoB^1^*^00/100^ (*n* = 7) mice as well as aged Chr4^Δ70/Δ70^ (*n* = 6) and *Ldlr*^−/−^*ApoB^1^*^00/100^ (*n* = 8) mice. Prior to testing, the mice were fasted for 4 h in the morning, after which their fasting blood glucose was measured from the tail vein by using an Ascencia Elite XL glucose meter (Bayer, Leverkusen, Germany). After that, 0.25 U/kg of fast acting insulin (Actrapid^®^ Penfill^®^ 100 IU/mL, Novo Nordisk, Copenhagen, Denmark) was injected to the mice i.p. Their blood glucose was then recorded from the tail vein at 15, 30, 45, and 90 min after the injection.

A glucose tolerance test (GTT) was performed to young Chr4^Δ70/Δ70^ (*n* = 4) and *Ldlr*^−/−^*ApoB^1^*^00/100^ (*n* = 4) and aged Chr4^Δ70/Δ70^ (*n* = 8) and *Ldlr*^−/−^*ApoB^1^*^00/100^ (*n* = 6) mice. The mice were fasted for 4 h in the morning. An amount of 1 g/kg of D-glucose was then injected i.p. to the mice, and their blood glucose was recorded from the tail vein, similarly to the ITT.

Insulin secretion was measured from the young *Ldlr*^−/−^*ApoB^1^*^00/100^ (*n* = 4) and Chr4^Δ70/Δ70^ (*n* = 4) mice, and it was performed along with the GTT. An amount of 5 μL of blood was collected from the tail vein both before and 15, 30, 60, and 90 min after the 1 g/kg D-glucose i.p. injection. Insulin was measured from the whole blood by using an ultra-sensitive mouse insulin ELISA (#90080, Crystal Chem, Elk Grove Village, IL, USA) with a modified protocol [28].

### 2.3. Blood Glucose, Cell Count, and Lipid Analysis

Blood values of the mice were measured at the end of the study. The mice were fasted for 4 h prior to the sampling, and blood glucose was measured from the tail vein by using an Ascencia Elite XL glucose meter (Bayer, Leverkusen, Germany). For blood count and plasma lipid analysis, the mice were sacrificed with CO_2_ and the whole blood was collected into EDTA micro tubes via cardiac puncture. Blood count was recorded from the EDTA whole blood by an animal diagnostic laboratory Movet Oy (Kuopio, Finland). For lipid analysis, EDTA plasma was separated and stored in −70 °C until the analysis. All the lipid analyses were performed in Wihuri Research Institute (Helsinki, Finland). Total cholesterol and triglyceride levels were analyzed by using Thermo Fisher Scientific (Waltham, MA, USA) analysis kits 981813 and 981301. Plasma lipoprotein profiles were determined with Superose 6 Increase 10/300 GL column, with 25 µL plasma.

### 2.4. Metabolic Rate

The basal metabolic rate of the mice was measured by using PhenoMaster Next Generation Home Cage Phenotyping platform (TSE systems, Inc., Berlin, Germany) including four individual PhenoMaster cages with automatic sensors for temperature, O_2_, CO_2_, and motion. The measurement was performed using randomly selected *Ldlr*^−/−^*ApoB^1^*^00/100^ (*n* = 4) and Chr4^Δ70/Δ70^ (*n* = 4) mice at 6 months of age. The mice were individually housed in the PhenoMaster home cages for 72 h in total. The mice acclimatized to the new cages for 24 h, after which the actual measurements started. For the following 48 h, energy and water intake (*ad libitum*), activity, O_2_ consumption, and CO_2_ production of the mice were automatically recorded once per hour. Calculations for heat production and respiratory exchange ratio (RER) were performed according to the PhenoMaster operating instruction manual for calorimetry, based on Weir 1949 [29]. Averages for all the parameters were then calculated separately for dark and light hours. The data were normalized to lean body mass of each mouse obtained from MRI (described below), as recommended by the manufacturer.

Prior to MRI, the mice were anesthetized with an inhalation gas mixture consisting of 5% isoflurane, 70% N2, and 30% O_2_. Anesthesia was maintained with isoflurane levels 1.5–2.5%, and the body temperature of the mice was sustained with a warm water pad. Respiration was monitored with a pneumatic pillow and a Model 1025 monitoring system (Small Animal Instruments Inc., Stony Brook, NY, USA). To determine the lean body mass of the mice, the mice were imaged in vivo with horizontal 7 T magnet (Bruker BioSpin, Ettlingen, Germany) using a quadrature volume coil controlled by a Bruker console (ParaVision 6.0.1, Bruker GmbH, Ettlingen, Germany) with the DIXON technique, which produces two images separating the signals from fat and water content [30]. The lean body mass was then estimated based on the fat mass which was calculated by utilizing fat-only images and the bodyweight of the mice. The Fast Low Angle Shot (FLASH) gradient echo sequence was used as a readout sequence. The following imaging parameters were used: echo time TE = 4.2 ms, repetition time TR = 40 ms, and resolution 480 × 120 × 88. The size of the field of view (FOV) was adjusted separately for every measurement. Additionally, the acquisition was gated prospectively according to the respiratory motion. All MRI analyses were performed with MATLAB version R2022a (MathWorks Inc., Natick, CA, USA) and Aedes Software 1.0 (http://aedes.uef.fi, accessed on 3 of August 2021).

### 2.5. Histology

For histopathological analysis, mouse tissue samples were harvested right after the sacrification and placed in 4% formaldehyde-Phosphate-Buffered Saline (pH 7.4) overnight. The samples were then processed into paraffin and cut into 4 µm cross-sections. For basic histopathology, the tissue sections were stained with hematoxylin-eosin. Pancreatic α- and β-cells were immunostained with primary antibodies against glucagon (Dako A0565, Rabbit anti-human glucagon, Agilent, Santa Clara, CA, USA) and insulin (Dako A0564, Guinea pig anti-insulin, Agilent, Santa Clara, CA, USA) and appropriate fluorescent secondary antibodies (for glucagon, A21442, chicken anti-rabbit A594, Thermo Fisher Scientific, Waltham, MA, USA, and for insulin, BA-7000 Goat anti-guinea pig with A-2011 Fluorescein Avidin DCS, Vector laboratories, Newark, CA, USA). For pancreatic islet cell proliferation, Ki-67 primary antibody (ab15580, Abcam, Cambridge, UK) and biotinylated goat anti-rabbit secondary antibody (BA-1000, Vector laboratories, Newark, CA, USA) were used. The stained sections were then imaged with a microscope (ECLIPSE Ni-E, Nikon instruments Inc., Tokyo, Japan) and analyzed with Fiji software version 2.3.0 [31]. For adipocyte and pancreatic islet size analysis, the region of interest (ROI) was drawn following the outlines of adipocytes or islets, and their area was measured. For the white adipose inflammatory cells, the ROI was drawn around the inflammatory cell clusters, and their area was presented as a percentage of the whole section. For the liver lipid content and pancreatic α- and β-cell content and proliferation analysis, the Fiji color threshold tool was used, and the area of interest was detected by its color and presented as a percentage of the image area or ROI.

### 2.6. Cell Culture and Seahorse

To study the role of ANRIL in human hepatocyte metabolism, human hepatoblastoma (HepG2) cells were first cultured in Minimum Essential Medium (Gibco™, #11095-080, Thermo Fisher Scientific, Waltham, MA, USA) with added 10% FBS, 1% Penicillin-Streptomycin, 1% MEM Non-Essential Amino Acids (100×), 1% sodium pyruvate (100 mM), and 1% GlutaMAX. Then, to accomplish downregulation of ANRIL, 100,000 cells/well were plated on 12-well plates and transfected either with 10 nM ANRIL Dicer-Substrate siRNA (DsiRNA, hs.Ri.CDKN2B-AS1.13.1., Integrated DNA Technologies, Coralville, IA, USA) or negative control DsiRNA (DS NC1 negative control duplex, Integrated DNA Technologies, Coralville, IA, USA) or left non-treated for 48 h. Transfections were made by using Minimum Essential Medium with DharmaFECT3 Transfection reagent (Dharmacon Reagents, Horizon Discovery Ltd., Cambridge, UK) in a 2 μL/well concentration. After the transfection, the HepG2 cells were lifted with trypsin and continued with a transfection efficiency analysis (described in Section 2.7) or mitochondrial function measurement.

For mitochondrial function and cell metabolic characterization, 10,000 ANRIL DsiRNA (*n* = 5), negative control DsiRNA (*n* = 6), and non-treated (*n* = 6) HepG2 cells were transferred onto s Seahorse XF Cell Mito Stress Test Kit 96-well plate (Agilent Technologies, Santa Clara, CA, USA). After that, the basal, ATP-linked, and maximal oxygen consumption rates (OCR) of the live cells were measured by using a Seahorse XFe96 Analyzer (Agilent) with automated timely injections of Oligomycin, Carbonyl cyanide-p-trifluoromethoxyphenylhydrazone (FCCP), and rotenone and antimycin A. Oligomycin was injected after the basal measurement, and it inhibits ATP synthase, resulting in decreased mitochondrial respiration, representing the portion of respiration being used to drive ATP production [32,33]. The second injection with FCCP disturbs the mitochondrial membrane potential and results in maximal oxygen consumption by Electron Transport Chain (ETC) complex IV. Finally, the ETC complexes I and III are inhibited by the injection of rotenone and antimycin, shutting down mitochondrial respiration, and non-mitochondrial respiration is measured. After the analysis, the cells were lysed with RIPA Lysis buffer, proteins were extracted with the BCA Protein Assay Kit (Pierce™, Waltham, MA, USA), and the total protein concentration of each well was measured with a microplate reader (CLARIOstar^®^, BMG Labtech, Ortenberg, Germany). Finally, the OCR was normalized to the sample protein concentration, and the data were analyzed by using Seahorse Wave Desktop Software version 2.6 (Agilent Technologies, Santa Clara, CA, USA).

### 2.7. Gene Expression Analysis

Gene expression analyses were performed from snap-frozen mouse tissues stored in −70 °C. First, total RNA was extracted from tissues with TRI Reagent™ Solution (Invitrogen™, Thermo Fisher Scientific, Waltham, MA, USA) and from HepG2 cells by using the RNeasy Mini Kit (Qiaqen, Hilden, Germany). The RNA obtained from tissues was treated with DNA-free™ DNA Removal Kit (Invitrogen™, Thermo Fisher Scientific, Waltham, MA, USA), and for cultured cells, RNase-Free Dnase Set (Qiaqen, Hilden, Germany) was used. The RNA was then reverse-transcribed into cDNA using the RevertAid First Strand cDNA Synthesis Kit (Invitrogen™, Thermo Fisher Scientific, Waltham, MA, USA) and random hexamer primers (Promega, Madison, WI, USA). Gene expression was measured with quantitative Polymerase Chain Reaction (qPCR) (StepOnePlus™ Real-Time PCR System, Applied Biosystems, Thermo Fisher Scientific, Waltham, MA, USA), by using the TaqMan™ Universal PCR Master Mix (Applied Biosystems, Thermo Fisher Scientific, Waltham, MA, USA) and TaqMan-based assays from Integrated DNA Technologies (Coralville, IA, USA) and Thermo Fisher Scientific (Waltham, MA, USA) (Supplementary Table S1). The measured mRNA levels were normalized to endogenous control *Gapdh*, *Rn18s* or *Ppia*, and relative gene expression levels were analyzed by using 2–∆∆Ct method [34].

The ANRIL DsiRNA transfection efficiency in the HepG2 cells was measured from the RNA by one-step reverse transcription ddPCR by using TaqMan-based assays (Integrated DNA Technologies, Coralville, IA, USA) for ANRIL exons 5–6 and the *HPRT1* housekeeper gene (Supplemental Table S1). One-Step RT-ddPCR Advanced Kit for Probes (Bio-Rad laboratories, Hercules, CA, USA) components were combined with the assays and the RNA template according to the manufacturer’s instructions. The droplets were generated with the QX200™ Automated Droplet Generator using the Droplet Generator Oil for Probes (Bio-Rad laboratories, Hercules, CA, USA). Reverse transcription and amplification were performed with the C1000 Thermal Cycler (Bio-Rad laboratories, Hercules, CA, USA) with the following conditions: 60 min at 50 °C, 10 min at 95 °C, 40× (30 s at 95 °C, 1 min at 60 °C), and 10 min at 98 °C. The droplets were left to stabilize at 8 °C between 3 and 18 h, after which the results were read on the QX200 Droplet Reader (Bio-Rad) and analyzed using QX Manager Software version 2.1 (Bio-Rad laboratories, Hercules, CA, USA). The data were normalized to *HPRT1* and the average copy number (N/100 ng RNA) of DsiRNA treated samples were compared to negative control and untreated cells.

### 2.8. Statistical Analysis

All statistical analyses were performed by using GraphPad Prism version 9.1.0. (GraphPad Software LLC, Boston, MA, USA). Differences in means between the two groups was tested by using a two-tailed student-*t* test. When comparing more than two groups, the difference in means was tested by using one-way ANOVA or two-way mixed-effect ANOVA, with Tukey’s multiple comparisons test or Šídák’s multiple comparisons test. The number of replicates and the exact statistical methods used in each experiment are stated in each figure legend. For all the statistical analyses, the difference in means was considered statistically significant when *p* or adjusted *p* < 0.05. All charts represent the data as mean ± standard deviation (SD).

## 3. Results

### 3.1. Deletion of the 9p21.3 Risk Interval Ortholog Leads to Obesity and Enlargement of Adipocytes during Aging in Ldlr^−/−^ApoB^100/100^ Mice on a Standard Diet

Chr4^Δ70/Δ70^*Ldlr*^−/−^*ApoB*^100/100^ mice (*n* = 6) and *Ldlr*^−/−^*ApoB*^100/100^ control mice (*n* = 8) were aged on a standard murine laboratory diet for 6 months or for one year (Chr4^Δ70/Δ70^ *n* = 10; *Ldlr*^−/−^*ApoB*^100/100^ *n* = 8) (Figure 1A). At the age of 6 months, the body weight did not differ between Chr4^Δ70/Δ70^ and *Ldlr*^−/−^*ApoB*^100/100^ mice, but at the age of one year, the Chr4^Δ70/Δ70^ mice showed significantly increased body weight (36.16 ± 4.10 vs. 31.95 ± 2.99 g; *p* = 0.03) (Figure 1B). Moreover, the adipocyte size in Chr4^Δ70/Δ70^ mice was increased (5226 ± 1819 vs. 3230 ± 1276 μm; *p* = 0.02) in comparison to *Ldlr*^−/−^*ApoB*^100/100^ mice at one year but not in young mice (Figure 1C,D). Aging also increased lipid accumulation in the liver due to hypercholesterolemia, but Chr4^Δ70/Δ70^ did not have any effects on hepatic steatosis in young or aged mice (Figure 1E,F). The metabolic rate in the mice was recorded at the midpoint of the study (Figure 1A), and there were no statistically significant differences in the energy or water intake, activity, heat production, or respiratory exchange ratio (RER) between Chr4^Δ70/Δ70^ (*n* = 4) and *Ldlr*^−/−^*ApoB*^100/100^ mice (*n* = 4) (Supplemental Figure S1A–G). The values were normalized to the lean body mass of the mice. Nighttime energy intake and activity were significantly higher in Chr4^Δ70/Δ70^ mice compared to daytime, but there was no significant difference between the genotypes at daytime or nighttime food intake or activity (Supplemental Figure S1A–G). As we reported previously, in young Chr4^Δ70/Δ70^*Ldlr*^−/−^*ApoB*^100/100^ mice, plasma fasting glucose was significantly increased with no differences in the blood lipid parameters in comparison to *Ldlr*^−/−^*ApoB*^100/100^ mice [26]. In aged mice, however, plasma lipoprotein profile (Supplemental Figure S2A), fasting glucose, total cholesterol, triglycerides, and blood cell count were not affected by the Chr4^Δ70/Δ70^ (Table 1).

### 3.2. Chr4^Δ70/Δ70^ Leads to Inhibition of Sirt1-Ppargc1a-Ucp2 Axis and Promotes Inflammation in White Adipose Tissue in Ldlr^−/−^ApoB^100/100^ Mice

**Figure 2:**
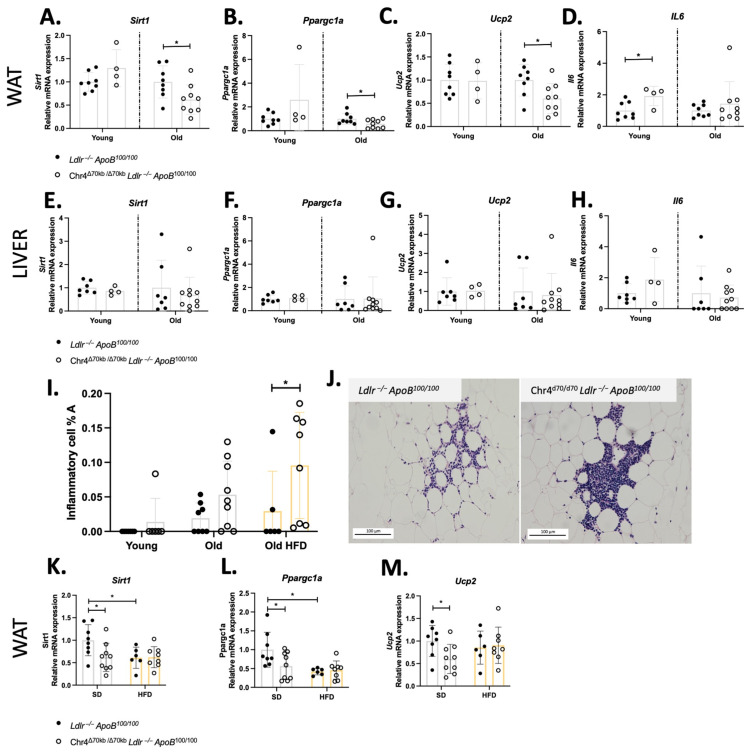
The *Sirt1-Ppargc1a-Ucp2* pathway is downregulated by *Chr4^Δ70/Δ70^* in the white adipose in aged mice. The mRNA expression of (**A**) *Sirt1*, (**B**) *Ppargc1a*, (**C**) *Ucp2*, and (**D**) *Il6* of young Chr4^Δ70/Δ70^ (*n* = 4) and *Ldlr*^−/−^*ApoB*^100/100^ (*n* = 8) and aged Chr4^Δ70/Δ70^ (*n* = 9) and *Ldlr*^−/−^*ApoB*^100/100^ mice (*n* = 8) white adipose tissue. The mRNA expression of (**E**) *Sirt1*, (**F**) *Ppargc1a*, (**G**) *Ucp2*, and (**H**) *Il6* in the liver of young Chr4^Δ70/Δ70^ (*n* = 4) and *Ldlr*^−/−^*ApoB*^100/100^ (*n* = 7) and old Chr4^Δ70/Δ70^ (*n* = 10) and *Ldlr*^−/−^*ApoB*^100/100^ (*n* = 7) mice. (**I**) Inflammatory cell percentage area in white adipose tissue of young Chr4^Δ70/Δ70^ (*n* = 6) and *Ldlr*^−/−^*ApoB*^100/100^ (*n* = 7) and aged Chr4^Δ70/Δ70^ (*n* = 9) and *Ldlr*^−/−^*ApoB*^100/100^ (*n* = 8) mice on SD and in response to HFD (Chr4^Δ70/Δ70^ *n* = 8, *Ldlr*^−/−^*ApoB*^100/100^ *n* = 6). (**J**) Representative image of inflammatory cell clusters in white adipose tissue of aged Chr4^Δ70/Δ70^ and *Ldlr*^−/−^*ApoB*^100/100^ mice. Scale bar is 100 µm. The expression of (**K**) *Sirt1*, (**L**) *Ppargc1a*, and (**M**) *Ucp2* in white adipose of aged Chr4^Δ70/Δ70^ (*n* = 8) and *Ldlr*^−/−^*ApoB*^100/100^ (*n* = 6) mice in response to HFD. The mRNA expression of target genes was normalized to endogenous control *rn18s.* Gene expression levels were analyzed separately for young and old mice by using 2–∆∆Ct method, and Chr4^Δ70/Δ70^gene expression is presented in relation to the *Ldlr*^−/−^*ApoB^1^*^00/100^ –group of the same age, and comparison between groups was performed by using a two-tailed *t*-test. For the adipose inflammatory cell area and old mice gene expression on SD and HFD, ANOVA with Šídák’s multiple comparisons and Tukey’s multiple comparisons test were used. Asterisk (*) indicates statistical significance. All differences were considered statistically significant when *p* or adjusted *p* < 0.05.

To further investigate white adipose metabolism, we measured the mRNA expression of the key regulator genes for metabolic homeostasis and oxidative stress. The gene expression analysis revealed no changes in mRNA expression of *Sirt1*, *Pgc1a*, and *Ucp2* in the white adipose tissue of young Chr4^Δ70/Δ70^ and *Ldlr*^−/−^*ApoB*^100/100^ mice, but the pathway was significantly downregulated by Chr4^Δ70/Δ70^ in aged mice (Figure 2A–C). Interestingly, young Chr4^Δ70/Δ70^ mice showed also significantly increased *Il6* expression in comparison to *Ldlr*^−/−^*ApoB^1^*^00/100^ in the white adipose tissue, while there was no difference between the genotypes in aged mice (Figure 2D). In the liver, the *Sirt1-Ppargc1a-Ucp2* axis or *Il6* were not affected by Chr4^Δ70/Δ70^ in young nor aged mice (Figure 2E–H).

When looking at the histopathology of the white adipose tissue, young mice did not show significant inflammatory cell infiltration regardless of the genotype (Figure 2I), while in aged mice, adipose inflammatory cells were present and organized as distinct clusters (Figure 2J). The percentage area of these clusters was higher in aged Chr4^Δ70/Δ70^ mice in comparison to *Ldlr*^−/−^*ApoB*^100/100^ mice, but the difference was not statistically significant (Chr4^Δ70/Δ70^ *n* = 9, *Ldlr*^−/−^*ApoB*^100/100^ *n* = 8; 0.05 ± 0.05 vs. 0.02 ± 0.02%; *p* = 0.09) (Figure 2J). To further investigate the inflammatory phenotype in white adipose tissue, we challenged the aged Chr4^Δ70/Δ70^ and *Ldlr*^−/−^*ApoB*^100/100^ mice with HFD for 3 months, to elevate pro-inflammatory activation and oxidative stress. HFD did not significantly affect to the body weight of the mice, but it further increased the size of adipocytes in both Chr4^Δ70/Δ70^ and *Ldlr*^−/−^*ApoB*^100/100^ mice (Supplemental Figure S2B,C). The size of adipocytes did not significantly differ between Chr4^Δ70/Δ70^ and *Ldlr*^−/−^*ApoB*^100/100^ mice after HFD. However, after HFD, the inflammatory cell area was significantly higher in the Chr4^Δ70/Δ70^ mice than in the *Ldlr*^−/−^*ApoB*^100/100^ mice (Chr4^Δ70/Δ70^ *n* = 8, *Ldlr*^−/−^*ApoB*^100/100^ *n* = 6; 0.10 ± 0.08 vs. 0.03 ± 0.06%; *p* = 0.042; Figure 2I). The *Sirt1-Ppargc1a-Ucp2* pathway was not affected by Chr4^Δ70/Δ70^ in HFD, yet the HFD itself seemed to downregulate the pathway in *Ldlr*^−/−^*ApoB*^100/100^ mice (Figure 2K–M). There were no differences in plasma lipoprotein profiles between the study groups (Supplemental Figure S2D).

### 3.3. Chr4^Δ70/Δ70^ Downregulates the Expression of Insulin Receptor Specifically in White Adipose Tissue in ApoB^100/100^ Mice

**Figure 3:**
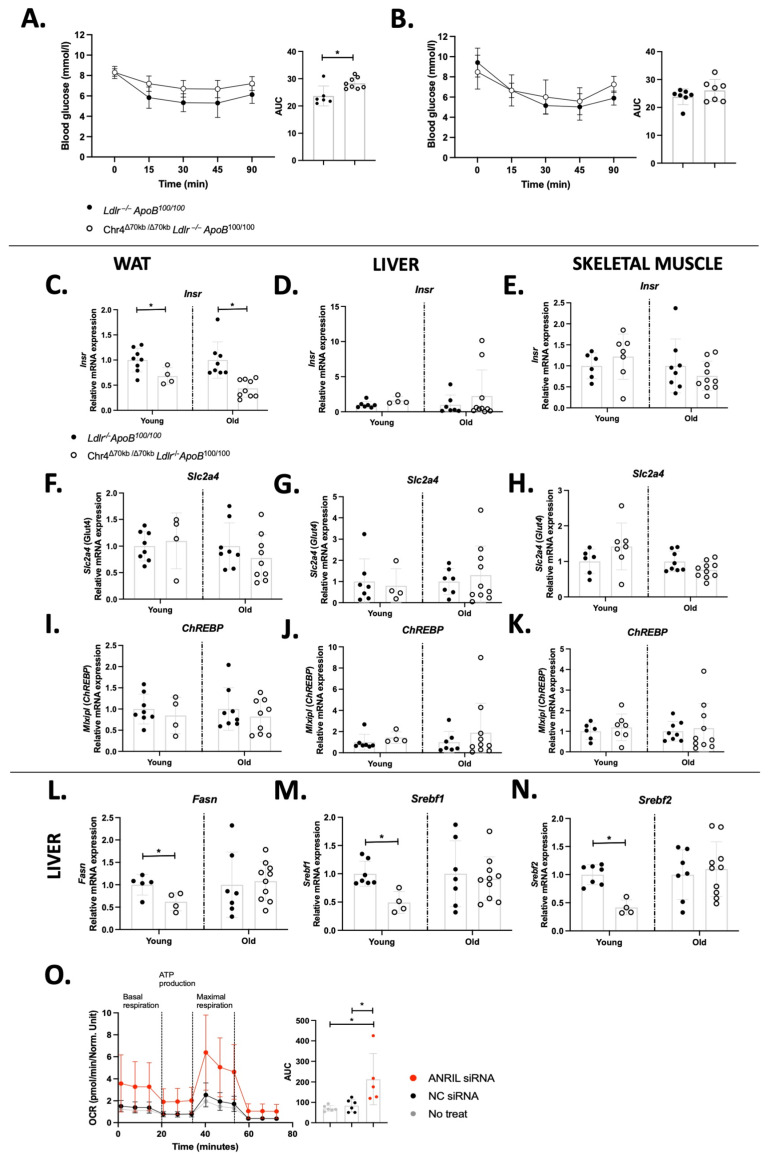
*Chr4^Δ70/Δ70^* causes insulin resistance and decreased insulin receptor expression in a white adipose tissue-specific manner. (**A**) Insulin tolerance of aged Chr4^Δ70/Δ70^ (*n* = 8) and Ldlr^−/−^ApoB^100/100^ (*n* = 6) and (**B**) young Chr4^Δ70/Δ70^ (*n* = 7) and Ldlr^−/−^ApoB^100/100^ (*n* = 7) mice after 4 h of fasting. Relative Insr mRNA expression in (**C**) white adipose, (**D**) the liver and (**E**) skeletal muscle in young Chr4^Δ70/Δ70^ (*n* = 4–7) and Ldlr^−/−^ApoB^100/100^ (*n* = 6–8) and aged Chr4^Δ70/Δ70^ (*n* = 9–10) and Ldlr^−/−^ApoB^100/100^ (*n* = 7–8) mice. Relative Scl2a4 mRNA expression in (**F**) white adipose, (**G**) the liver and (**H**) skeletal muscle of young Chr4^Δ70/Δ70^ (*n* = 4–7) and Ldlr^−/−^ApoB^100/100^ (*n* = 6–8) and aged Chr4^Δ70/Δ70^ (*n* = 9–10) and Ldlr^−/−^ApoB^100/100^ (*n* = 7–8) mice. Relative ChREBP mRNA expression in (**I**) white adipose, (**J**) the liver and (**K**) skeletal muscle of young Chr4^Δ70/Δ70^ (*n* = 4–7) and Ldlr^−/−^ApoB^100/100^ (*n* = 6–8) and aged Chr4^Δ70/Δ70^ (*n* = 9–10) and Ldlr^−/−^ApoB^100/100^ (*n* = 7–8) mice. Relative (**L**) Fasn, (**M**) Srebf1 and (**N**) Srebf2 mRNA expression in the liver of young Chr4^Δ70/Δ70^ (*n* = 4) and Ldlr^−/−^ApoB^100/100^ (*n* = 5–7) and aged Chr4^Δ70/Δ70^ (*n* = 10) and Ldlr^−/−^ApoB^100/100^ (*n* = 7) mice. (**O**) Oxygen consumption rate (OCR) of HEPG2 cells after transfection with ANRIL (*n* = 5) or negative control (NC) (*n* = 6) DsiRNA or without treatment (*n* = 6). Data are normalized to the total protein content of each sample. For graphs (**A**,**B**,**O**), the area under the curve (AUC) was determined for each individual and difference in means between the two groups was measured with *t*-test, and for graph (**O**), with one-way ANOVA and Tukey’s multiple comparison test. mRNA levels were normalized to rn18s and analyzed separately for young and old mice by using the 2–∆∆Ct method. Chr4^Δ70/Δ70^ group expression levels are presented in relation to Ldlr^−/−^ApoB^100/100^ of the same age, and comparison was performed with *t*-test. Asterisk (*) indicates statistical significance. Differences were considered statistically significant when *p* or adjusted *p* < 0.05.

In addition to increased bodyweight and adipocyte enlargement, aged Chr4^Δ70/Δ70^ mice (*n* = 8) showed an impaired response to insulin in comparison to *Ldlr*^−/−^*ApoB*^100/100^ mice (*n* = 6) (AUC 28.33 ± 2.08 vs. 23.68 ± 3.67; *p* = 0.01) (Figure 3A), while in young mice there were no significant differences between the genotypes (Figure 3B). Moreover, Chr4^Δ70/Δ70^ did not alter insulin response on HFD in *Ldlr*^−/−^*ApoB*^100/100^ mice (Supplemental Figure S2E). However, there was no difference in the glucose tolerance between young Chr4^Δ70/Δ70^ (*n* = 4) and *Ldlr*^−/−^*ApoB*^100/100^ (*n* = 4) mice (Supplemental Figure S2F), but in aged Chr4^Δ70/Δ70^ (*n* = 8) mice, the response to glucose was slightly more efficient than in *Ldlr*^−/−^*ApoB*^100/100^ (*n* = 6) (Supplemental Figure S2G).

The effects of Chr4^Δ70/Δ70^ on the expression of insulin receptors (*Insr*) and glucose transport receptors in white adipose tissue, the liver, and skeletal muscle were measured at the mRNA level in young and aged Chr4^Δ70/Δ70^ and *Ldlr*^−/−^*ApoB^1^*^00/100^ mice. Both young and aged Chr4^Δ70/Δ70^ mice showed a significant downregulation of *Insr* expression in white adipose tissue, but not in the liver or skeletal muscle (Figure 3C–E). However, the expression of the glucose transporter *Glut4*/*Slc2a4* (Figure 3F–H) and glucose-activated carbohydrate-responsive element-binding protein (*ChREBP*) (Figure 3I–K) did not differ between Chr4^Δ70/Δ70^ and *Ldlr*^−/−^*ApoB*^100/100^ mice in white adipose tissue, skeletal muscle, or the liver in either age group.

Cellular cholesterol and fatty acid synthesis are regulated in a response to insulin via *Srebp* genes, *Sreb1* and -*2*, and a master regulator of fatty acid synthesis, *Fasn*. Hepatic expression of all these genes was downregulated by Chr4^Δ70/Δ70^ in young mice but was not affected in aged mice (Figure 3L–N). This prompted us to study the role of ANRIL deficiency in the metabolic characteristics in a hepatocyte cell line. ANRIL expression was knocked down in the HepG2 cells by DsiRNA transfection, and the extracellular flux and mitochondrial function were analyzed by using Seahorse analyzer. A 62% downregulation of ANRIL was confirmed in ANRIL DsiRNA-treated cells in comparison to non-treated cells, and a 42% downregulation was confirmed in comparison to negative control (NC) DsiRNA. The downregulation of ANRIL significantly increased the oxygen consumption rate (OCT) of HepG2 at the basal level, but also the oligomycin-induced ATP production related respiration, FCCP-related maximal respiration, and non-mitochondrial ROT+AA-induced respiration were increased by ANRIL knockdown in comparison to non-treated (AUC 213.30 ± 124.90 vs. 70.44 ± 14.04; *p* = 0.01) and negative control DsiRNA transfected hepatocytes (AUC 213.30 ± 124.90 vs. 84.14 ± 31.11; *p* = 0.02) (Figure 3O). This indicates higher oxidative phosphorylation (OXPHOS) and ATP production by the ANRIL downregulation in hepatocytes.

### 3.4. Chr4^Δ70/Δ70^ Reduces Cell Proliferation in Pancreatic Islets in Young Ldlr^−/−^ApoB^100/100^ Mice

**Figure 4:**
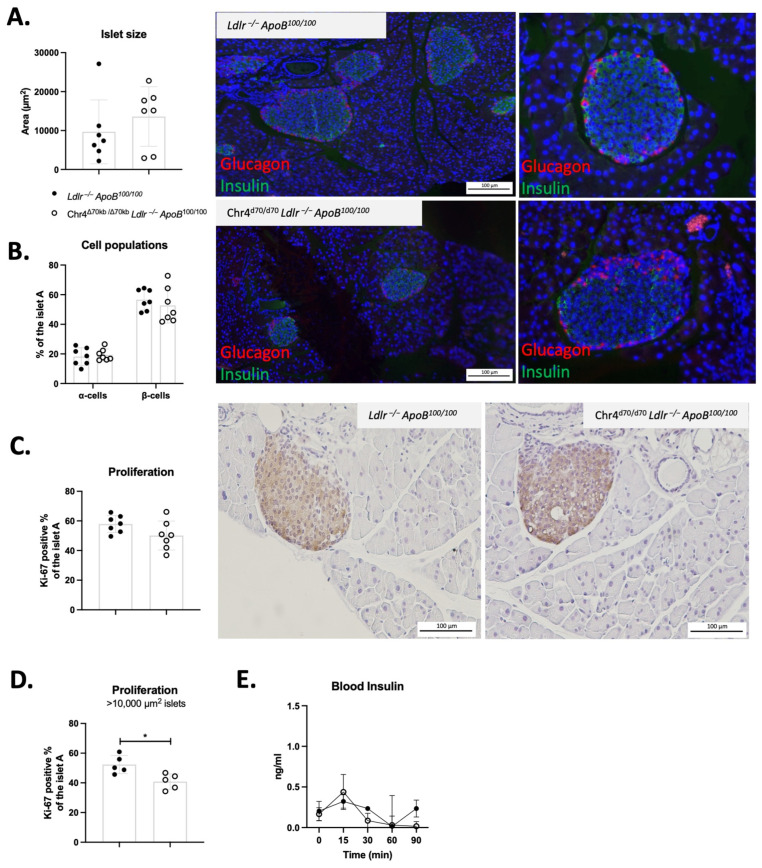
Chr4^Δ70/Δ70^ does not affect pancreatic β-cell number but may reduce the cell proliferation rate already in young *Ldlr*^−/−^*ApoB*^100/100^ mice. (**A**) Pancreatic islet size, (**B**) proportion of α- and β-cells, and (**C**) proliferation of mixed and (**D**) >10,000 μm^2^ pancreatic islets in young *Ldlr*^−/−^*ApoB*^100/100^ (*n* = 5–7) and Chr4^Δ70/Δ70^ mice (*n* = 5–7). Primary antibody against glucagon was used as a marker for the pancreatic α-cells, and insulin ab was used for the β-cells. For visualization, fluorescent secondary antibodies were used. In the representative figures, glucagon positive cells appear in red and insulin in green. Nuclei were counterstained with DAPI (blue). Ki-67 antibody was used as a marker of cell proliferation and visualized with DAB. (**E**) For the islet function, insulin secretion of *Ldlr*^−/−^*ApoB*^100/100^ (*n* = 4) and Chr4^Δ70/Δ70^ mice (*n* = 4) was measured both in fasted state (0 min) and in response to 1 g/kg i.p. glucose at time points 15, 30, 60 and 90 min after the administration. Asterisk (*) indicates statistical significance. Difference in mean between *Ldlr*^−/−^*ApoB*^100/100^ and Chr4^Δ70/Δ70^ mice was measured by using *t*-test, and it was considered statistically significant when *p* < 0.05.

To investigate whether the insulin-resistant phenotype of the aged Chr4^Δ70/Δ70^ mice could be preceded by dysfunction of insulin and glucagon signaling in the pancreatic islets, we examined the pancreatic islets and proportions of β- and α-cells in the young *Ldlr*^−/−^*ApoB*^100/100^ (*n* = 7) and Chr4^Δ70/Δ70^ mice (*n* = 7). There were no significant differences in the average islet size or the proportion of β- and α-cells per islet between Chr4^Δ70/Δ70^ and *Ldlr*^−/−^*ApoB*^100/100^ mice (Figure 4A,B). There was also no difference in the average proportion of proliferating cells as measured by Ki-67 staining of the islets (Figure 4C). However, there was great variation in the size of the islets, and when we sorted the islets by size, we found that in the large well-developed islets (>10 000 um^2^), the Ki-67 positive area was significantly reduced by Chr4^Δ70/Δ70^ (40.85 ± 5.17 vs. 52.27 ± 6.10%; *p* = 0.01) (Figure 4D). Nevertheless, there was no difference in the insulin secretion between young Chr4^Δ70/Δ70^ and *Ldlr*^−/−^*ApoB*^100/100^ mice (Figure 4E).

### 3.5. Chr4^Δ70/Δ70^ Downregulates Cdkn2b Specifically in White Adipose Tissue in Ldlr^−/−^ApoB^100/100^ Mice

**Figure 5:**
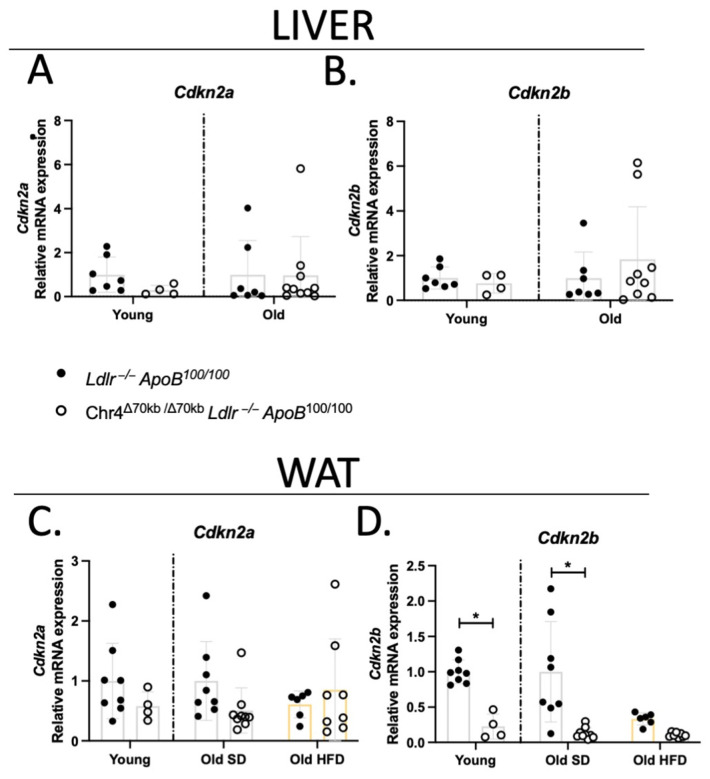
*Cdkn2b*, but not *Cdkn2a*, expression is downregulated by Chr4^Δ70/Δ70^ in the white adipose tissue of *Ldlr*^−/−^*ApoB*^100/100^ mice. Relative mRNA level of (**A**) *Cdkn2a* and (**B**) *Cdkn2b* in the liver of young Chr4^Δ70/Δ70^ (*n* = 4) and *Ldlr*^−/−^*ApoB*^100/100^ (*n* = 7) and aged Chr4^Δ70/Δ70^ (*n* = 9–10) and *Ldlr*^−/−^*ApoB*^100/100^ (*n* = 7) mice. Relative mRNA level of (**C**) *Cdkn2a* and (**D**) *Cdkn2b* in white adipose tissue of young Chr4^Δ70/Δ70^ (*n* = 4) and *Ldlr*^−/−^*ApoB*^100/100^ (*n* = 8), and aged Chr4^Δ70/Δ70^ (*n* = 9) and *Ldlr*^−/−^*ApoB*^100/100^ mice (*n* = 8), and in aged mice in response to three months HFD (Chr4^Δ70/Δ70^ *n* = 8, *Ldlr*^−/−^*ApoB*^100/100^ *n* = 6). Measured mRNA levels were normalized to endogenous control rn18s. Gene expression levels in the liver were analyzed separately for young and old mice by using the 2–∆∆Ct method, and the Chr4^Δ70/Δ70^ data are presented in relation to the *Ldlr*^−/−^*ApoB*^100/100^ –group of the same age. Difference in means between *Ldlr*^−/−^*ApoB*^100/100^ and Chr4^Δ70/Δ70^ within an age group was measured by using two-tailed *t*-test. For white adipose tissue data containing old mice on both SD and HFD, the gene expression of old mice is presented in relation to SD *Ldlr*^−/−^*ApoB*^100/100^–group, and two-tailed ANOVA with Tukey’s multiple comparisons test was used to determine the difference between genotypes and diets within the old mice. Asterisk (*) indicates statistical significance. All differences were considered statistically significant when *p* < 0.05.

The 9p21.3 locus contains the genes of two important cell-cycle regulators, *CDKN2A* and *CDKN2B*, with some of the T2D and CAD risk SNPs having been associated with changes in the expression of these genes [35,36,37]. *Cdkn2a* and *Cdkn2b* are also present in the murine ortholog of the 9p21 locus, and thus, we analyzed the effect of HFD and Chr4^Δ70/Δ70^ on the expression of *Cdkn2a*/*b* in the liver and white adipose tissue of the *Ldlr*^−/−^*ApoB*^100/100^ mice. The expression of both *Cdkn2a* and *-b* remained unchanged by Chr4^Δ70/Δ70^ in the liver, in both young and aged mice (Figure 5A,B). Similarly, *Cdkn2a* was not affected by Chr4^Δ70/Δ70^ in the white adipose tissue, regardless of the age or diet (Figure 5C). However, *Cdkn2b* was significantly downregulated in the adipose tissue of Chr4^Δ70/Δ70^ mice, in both young (Chr4^Δ70/Δ70^ *n* = 4, *Ldlr*^−/−^*ApoB*^100/100^ *n* = 8; *p* = 0.01) and aged (Chr4^Δ70/Δ70^ *n* = 9, *Ldlr*^−/−^*ApoB*^100/100^ *n* = 8; *p* < 0.0001) mice on a standard diet (Figure 5D). In addition, HFD downregulated the expression of *Cdkn2b* in *Ldlr*^−/−^*ApoB^100/100^* mice in comparison to SD (*p* = 0.01), while the low levels of expression in the Chr4^Δ70/Δ70^ mice appeared not to be further influenced by the diet.

## 4. Discussion

The mechanisms of 9p21.3 genetic variation and ANRIL lncRNA in different cell types relevant to atherosclerosis have been slowly unveiling over the past decade, but their role in metabolism has been unclear. Variation in the 9p21.3 noncoding region has been associated with CAD and T2D, but the specific SNPs and their associations are distinct from each other [10,38,39]. However, obesity is a well-recognized risk factor for both CAD and T2D, and disruptions in metabolic homeostasis and DNA methylation of the ANRIL promoter region at birth has been associated with higher adiposity later in life [40]. In our previous study, we showed that the deletion of the 9p21.3 CAD risk locus orthologue (Chr4^Δ70/Δ70^) in *Ldlr*^−/−^*ApoB*^100/100^ mice promoted atherosclerosis in response to a prolonged HFD, but not a standard diet, due to enhanced macrophage pro-inflammatory activity [26]. We also demonstrated the mouse lncRNA Ak148321 forms circular splicing isoforms and is partially modulated by HFD.

Our current study demonstrates increased body weight by enhanced lipid accumulation and enlargement of adipocytes in aged Chr4^Δ70/Δ70^*Ldlr*^−/−^*ApoB*^100/100^ female mice on a standard laboratory diet. Additionally, the elevated lipid accumulation was not detected in the liver, indicating adipose tissue-specific mechanisms by the locus. As has been reported before, 9p21.3 variants do not consistently affect the traditional cardiometabolic risk factors like plasma fasting glucose, cholesterol, triglyceride, and lipoprotein levels in humans [15,16,17]. The present study also concluded that the lipid accumulation in white adipose tissue was not caused by increased calory intake, activity, or elevated plasma lipid levels, as they remained unchanged by Chr4^Δ70/Δ70^. In general, male mice are known to be more susceptible to diet-induced obesity than female mice [41,42]. However, we were encouraged to study the metabolic phenotype in females, as we had previously reported the elevated plasma fasting glucose and relative increase in body weight gain (%) in female Chr4^Δ70/Δ70^ mice on a standard diet, suggesting dysregulation of glucose metabolism or insulin signaling by Chr4^Δ70/Δ70^ [26].

Sirtuins, especially SIRT1, are expressed in metabolic tissues and are the key regulators of cellular metabolic rate. In white adipose tissue, SIRT1 deacetylates a range of substrates, including PGC1α, UCP2, and NFκB, and regulates the activity of PPARγ [43]. SIRT1 regulates glucose homeostasis and insulin secretion both directly and indirectly, as it influences adipogenesis and fat storage in white adipose tissue, which reflects into insulin signaling and secretion, but SIRT1 also regulates the volume of pancreatic β-cells [44]. Here, we found that Chr4^Δ70/Δ70^ reduced the expression of *Sirt1*, *Pgc1a*, and *Ucp2* in the white adipose tissue of aged *Ldlr*^−/−^*ApoB*^100/100^ mice. The expression of the *Sirt1-Pgc1a-Ucp2* axis remained unchanged in the liver, indicating adipose tissue-specific regulation. Reduced expression levels of *PGC1a*, *SIRT1*, and *UCP2* have been reported in obese individuals in human populations [45,46]. The downregulation of *PGC1a* and *SIRT1* impairs adipocyte differentiation and promotes the dysfunction of adipocytes. In addition, *SIRT1* and *UCP2* also mediate oxidative stress- and inflammation-related mechanisms [47,48]. In our study, the histological analysis of the white adipose tissue revealed minimal infiltration of inflammatory cells in young mice, but the infiltration increased with age. When we further promoted a pro-inflammatory phenotype with three months of HFD, we found that Chr4^Δ70/Δ70^ significantly increased the inflammatory cell area in the white adipose tissue. We also found elevated *Il-6* expression in white adipose tissue of the young Chr4^Δ70/Δ70^ mice, suggesting pro-inflammatory activity, though this difference was not observed in the aged mice. As expected, the expression of *Pgc1a* and *Sirt1* was downregulated by HFD in *Ldlr*^−/−^*ApoB*^100/100^ control mice. Interestingly, compared to the downregulation seen on SD, no additional changes were induced by the Chr4^Δ70/Δ70^ on HFD, bringing the expression levels of both groups on an HFD to equal levels. We did not detect any changes in body weight or insulin tolerance between Chr4^Δ70/Δ70^ and *Ldlr*^−/−^*ApoB*^100/100^ mice on HFD, likely due to our control mice also developing insulin resistance and significant obesity in response to an HFD. Nevertheless, these data demonstrate that the inhibition of the *Sirt1-Pgc1a-Ucp2* axis by Chr4^Δ70/Δ70^ in the aged mice on a standard diet likely predisposes the mice to elevated inflammation in white adipose tissue, which is a well-recognized risk factor for cardiometabolic disorders.

In line with obesity and reduced expression of the *Sirt1-Pgc1a-Ucp2* axis, we found that Chr4^Δ70/Δ70^ mice also developed insulin resistance during aging. Moreover, the white adipose tissue seemed to be the most insulin-resistant organ in the Chr4^Δ70/Δ70^ mice, as the expression of *Insr* was reduced only in the white adipose tissue and not in the liver or skeletal muscle. Interestingly, the genes regulating glucose transport and glucose metabolism, *Glut4* and *ChRebp*, remained unchanged in all the examined tissues regardless of the group. Moreover, glucose tolerance of the Chr4^Δ70/Δ70^ mice was not impaired, indicating the sustained function of glucose uptake both in the liver and skeletal muscle. Expression of *Insr* was already downregulated in young Chr4^Δ70/Δ70^ mice, before the increase in body weight or insulin resistance was observed. This suggests that dysregulated insulin signaling could be the predisposing factor leading to the inhibition of the *Sirt1-Pgc1a-Ucp2* axis, adipocyte dysfunction, and the eventual development of obesity and insulin resistance with age. Indeed, in humans, the downregulation of *INSR* in adipose tissue is an early molecular event which has been recognized to contribute to the development of adipocyte dysfunction and, eventually, systemic insulin resistance [49]. However, more research on the insulin signaling pathway is needed to conclusively demonstrate the mechanisms of Chr4^Δ70/Δ70^ in the regulation of insulin sensitivity in white adipose tissue.

Both impaired sirtuin and insulin signaling are known to downregulate liver cholesterol and fatty acid synthesis [50,51]. Already at a young age, the Chr4^Δ70/Δ70^ mice showed reduced expression of *Fasn*, *Srebp1*, and -*2*, which might have been directly or indirectly caused by impaired insulin or sirtuin signaling. To study the hepatocyte metabolic rate in more detail, we did an extracellular flux analysis of human hepatocytes together with ANRIL knockdown (KD). We found that the downregulation of ANRIL increased oxidative metabolism, respiratory capacity, and ATP production of the HepG2 cells. Oxidative phosphorylation is known to be upregulated in obesity and insulin resistance, both in humans and in mice, and it has been proposed to promote the disease phenotype by elevated production of reactive oxygen species (ROS) [52,53]. Elevated oxidative metabolism observed already at the basal level in ANRIL KD hepatocytes may indicate ROS-mediated mechanisms that could promote the systemic metabolic phenotype.

As the 9p21.3 risk locus has been linked to pancreatic β-cell proliferation, we also investigated if the metabolic phenotype could be partially explained by dysregulated pancreatic cell function. We analyzed the size of the Langerhans islets and their proportion of α- and β-cells in young Chr4^Δ70/Δ70^ and *Ldlr*^−/−^*ApoB*^100/100^ mice. However, we did not see any effect by Chr4^Δ70/Δ70^ on the size of the islets or the number of α- and β-cells. We also performed Ki67 immunohistochemical staining to detect proliferating cells in the pancreas. As the size of the islets varied vastly, we classified the islets according to their size and found that the Chr4^Δ70/Δ70^ mice showed a reduced Ki67-positive staining area in the large, >10,000 µm^2^ pancreatic islets, indicating reduced proliferative activity. Though most of the cells in the islets are β-cells, it is not possible to say whether the proliferation was reduced in both α- and β-cells. However, it seems the functionality of the islets was not affected as there were no differences in the insulin secretion in response to glucose. In human studies, the carriers of a T2D risk allele rs564398 (T) in ANRIL exon 2 had elevated ratios of circular to linear ANRIL, which was associated with decreased β-cell proliferation [21]. It is known that aging alone reduces β-cell proliferation in both humans and mice, and obesity and pre-diabetic conditions may also alter β-cell proliferation and volume; this would be in line with the obese and insulin-resistant phenotype in the Chr4^Δ70/Δ70^ mice [54].

The *CDKN2A/B* locus has been reported to have several metabolic effects and also to regulate the proliferation of adipocytes and pancreatic β-cells [55]. However, the specific effects of the risk haplotypes on the expression of CDKN2A/B and ANRIL remain somewhat unclear. Studies have reported both reduced, increased, as well as unaltered expression in association with the CAD and T2D risk SNPs [35,36,37]. In mice, Visel et al. reported reduced cardiac expression of both *Cdkn2a* and *-b* by the Chr4^Δ70/Δ70^ in wild-type backgrounds [13]. In the present study with our hyperlipidemic mouse model, the expression of *Cdkn2a* in the liver and white adipose tissue remained unchanged in both young and aged Chr4^Δ70/Δ70^ and *Ldlr*^−/−^*ApoB*^100/100^ mice on a standard diet, as well as in response to an HFD in the white adipose tissue of aged mice. However, the expression of *Cdkn2b* was downregulated by Chr4^Δ70/Δ70^ specifically in the white adipose tissue of both young and aged mice. In addition, the expression of *Cdkn2b* was significantly downregulated by an HFD, resulting in similar levels of expression in the Chr4^Δ70/Δ70^ and *Ldlr*^−/−^*ApoB*^100/100^ mice after the three months on an HFD. This could possibly explain the white adipose tissue-specific insulin resistance and the lipid accumulation caused by Chr4^Δ70/Δ70^. The reduced expression of *Cdkn2b* in *Ldlr*^−/−^*ApoB*^100/100^ control mice by an HFD may also explain why the phenotype of Chr4^Δ70/Δ70^ mice was not further altered by an HFD. Overall, it seems that in addition to aging and diet influencing the expression of *Cdkn2a/b*, the effect of Chr4^Δ70/Δ70^ varies depending on the tissue or cell type in question. This suggests that some elements contained in the deleted interval, such as the lncRNA Ak148321, are regulating these genes in tissue- and condition-specific manners.

## 5. Conclusions

Here, we have demonstrated that the reduced expression of insulin receptors in the white adipose tissue of young mice seems to lead, with aging, to insulin resistance and obesity in hyperlipidemic mice carrying a deletion of the murine 9p21.3 CAD risk locus orthologue. These results suggest that the 9p21.3 risk locus has a role in regulating white adipose tissue function, which likely increases the risk of T2D later in life. Potentially, the lncRNA ANRIL and its murine orthologue Ak148321, which are situated within the risk interval, could mediate these effects via regulation of the neighboring gene *Cdkn2b*, but further studies on the causality and effect of tissue-specific gene regulation are required.

## Figures and Tables

**Table 1:** Blood values of Chr4^Δ70/Δ70^ Ldlr^−/−^ApoB^100/100^ and Ldlr^−/−^ApoB^100/100^ mice.

Blood Values	Young Mice ^1^							Aged Mice							
	*Ldlr* ^−/−^ *ApoB* ^100/100^			Chr4^Δ70/Δ70^*Ldlr*^−/−^*ApoB*^100/100^			Ref.	*Ldlr* ^−/−^ *ApoB* ^100/100^				Chr4^Δ70/Δ70^*Ldlr*^−/−^*ApoB*^100/100^			
Glucose (mmol/L)	6.59	±	0.62 (n = 7)	7.35	±	0.58 (n = 6) * *p* = 0.046	(23)	7.36	±	0.43	(n = 8)	8.03	±	1.03	(n = 9)
Cholesterol (mmol/L)	7.47	±	1.60 (n = 8)	6.82	±	2.38 (n = 6)	(23)	6.98	±	1.14	(n = 8)	6.94	±	2.64	(n = 9)
Triglycerides (mmol/L)	1.37	±	0.52 (n = 8)	1.18	±	0.40 (n = 6)	(23)	1.68	±	0.42	(n = 8)	1.70	±	0.46	(n = 9)
WBCB (x10E09 cells/L)	11.96	±	4.69 (n = 7)	13.73	±	2.58 (n = 5)	(23)	14.91	±	12.17	(n = 8)	8.29	±	2.55	(n = 9)
%NEUT (%)	8.37	±	1.46 (n = 7)	8.56	±	2.14 (n = 5)	(23)	7.26	±	3.74	(n = 8)	9.42	±	1.77	(n = 9)
%LYM (%)	78.87	±	3.78 (n = 7)	80.52	±	6.35 (n = 5)	(23)	82.71	±	5.76	(n = 8)	80.19	±	3.15	(n = 9)
%MONO (%)	1.53	±	0.25 (n = 7)	2.02	±	0.85 (n = 5)	(23)	3.21	±	2.96	(n = 8)	3.40	±	1.11	(n = 9)
%EOS (%)	4.6	±	1.64 (n = 7)	2.52	±	0.76 (n = 5) * *p* = 0.026	(23)	3.81	±	1.96	(n = 8)	4.36	±	2.16	(n = 9)
%BASO (%)	6.27	±	3.80 (n = 7)	5.72	±	7.56 (n = 5)	(23)	2.61	±	5.59	(n = 8)	0.57	±	1.04	(n = 9)
%Retic (%)	0.39	±	0.11 (n = 7)	0.72	±	0.84 (n = 5)	(23)	4.28	±	1.64	(n = 8)	3.37	±	1.27	(n = 9)

^1^ Data of young mice has been previously published and is represented here for context and comparison. Total cholesterol and triglycerides were analyzed from EDTA plasma. Blood cell count analysis was performed from EDTA whole blood. Values are presented as average ± standard deviation. Difference in means between *Ldlr*^−/−^*ApoB^1^*^00/100^ and Chr4^Δ70/Δ70^ *Ldlr*^−/−^*ApoB^1^*^00/100^ groups within was tested by using *t*-test, and it was considered statistically significant when * *p* < 0.05.

## Data Availability

All the data presented are provided in the manuscript. Raw data are available on reasonable request from the corresponding author.

## Supplementary Materials

The following are available online at https://www.mdpi.com/article/10.3390/cells13110983/s1, Table S1: A list of pre-designed and custom qPCR and ddPCR assays used; Supplemental Figure S1: Basal metabolic rate of the mice was not affected by the Chr4^Δ70/Δ70^; Supplemental Figure S2: Chr4^Δ70/Δ70^ did not have effects on body weight gain, plasma lipoprotein profile, or insulin resistance after prolonged HFD in mice.
