# Supplementary material for: Deletion of the Murine Ortholog of the Human 9p21.3 Locus Leads to Insulin Resistance and Obesity in Hypercholesterolemic Mice
Source: Cells. 2024 Jun 5;13(11):983. doi: 10.3390/cells13110983 (PMC11171903; doi:10.3390/cells13110983)
Supplement: Supplementary file 1 [file cells-13-00983-s001.zip › cells-3006513-supplementary.pdf]

Supplemental Table S1. *A list of pre-designed and custom qPCR and ddPCR assays used.*

Species	Target	Manufacturer	Product code	
Mus musculus	*Cdkn2a*	Integrated DNA Technologies	Mm.PT.58.43961185	
Mus musculus	*Cdkn2b*	Integrated DNA Technologies	Mm.PT.58.7138437	
Mus musculus	*Fasn*	Integrated DNA Technologies	Mm.PT.58.14276063	
Mus musculus	*Il6*	Integrated DNA Technologies	Mm.PT.58.10005566	
Mus musculus	*Insr*	Integrated DNA Technologies	Mm.PT.58.9275253	
Mus musculus	*Sirt1*	Integrated DNA Technologies	Mm.PT.7263242	
Mus musculus	*Srebf1*	Thermo Fisher Scientific	Mm00550338_m1	
Mus musculus	*Srebf2*	Thermo Fisher Scientific	Mm01306293_m1	
Mus musculus	*Ppargc1a*	Integrated DNA Technologies	Mm.PT.58.17390716	
Mus musculus	*Mlxipl*	Integrated DNA Technologies	Mm.PT.56a.33592172	
Mus musculus	*Slc2a2*	Integrated DNA Technologies	Mm.PT.58.45872027	
Mus musculus	*Slc2a4*	Thermo Fisher	Mm0124550_m1	
Mus musculus	*Ucp2*	Integrated DNA Technologies	Mm.PT.58.11226903	
Homo sapiens	*HPRT1*	Integrated DNA Technologies	Hs.PT.58v.45621572	
**Custom assays**	**Target**	**Manufacturer**		
Homo sapiens	ANRIL Exon 5-6	Integrated DNA Technologies	Primer 1	5’-GCCACCAGATATATGTTATCTGTGCTTA-3’
			Primer 2	5’-TAGAAAGCAGTACTGACTCGGGAA-3’
			Probe	5’-/5SUN/AG CAT CAC T/ZEN/G TTA GGT GTG CTG GA/3IABkFQ/-3’

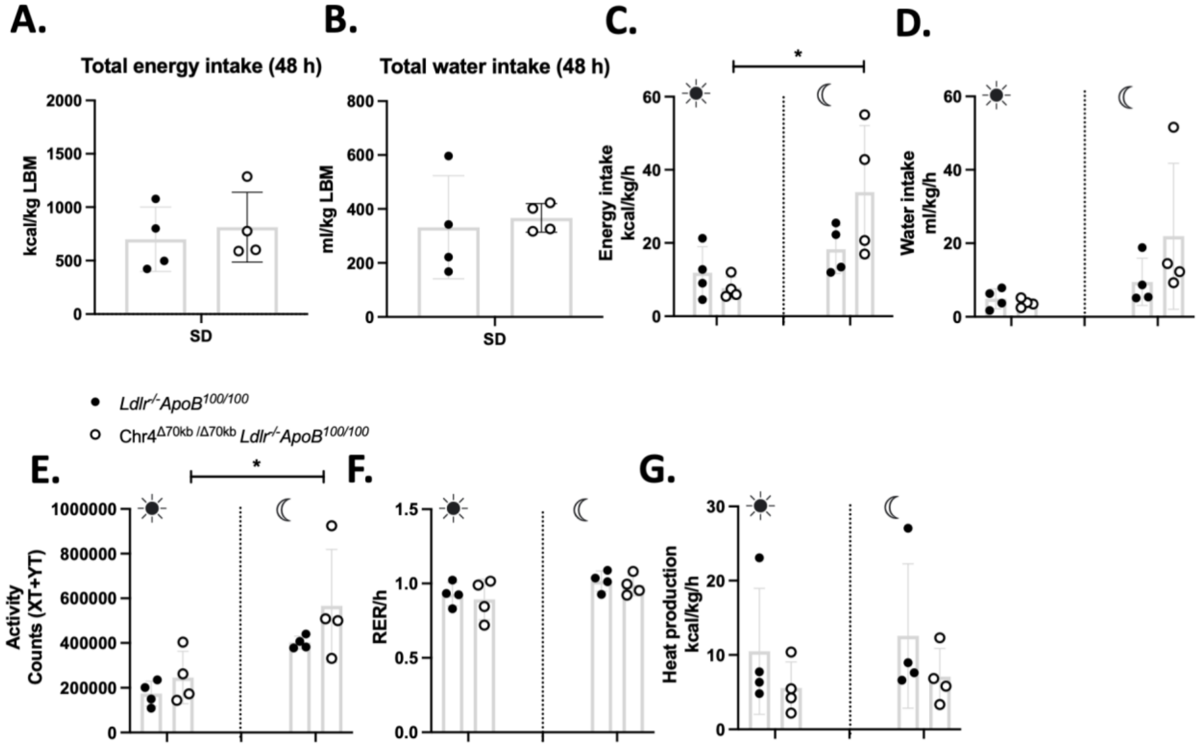

Supplemental Figure S1. ***Basal metabolic rate of the mice was not affected by the Chr4$^{\Delta70/\Delta70}$.***

A) Total 48 h energy and **B)** water intake, and average hourly **C)** energy intake, **D)** water intake, **E)** activity, **F)** heat production and **G)** respiratory exchange ratio (RER) of *Ldlr$^{-/-}$ApoB*$^{100/100}$ (n=4) and Chr4$^{\Delta70/\Delta70}$ (n=4) mice in light (☀) and dark (☾) hours over 48 hours follow up period.

RER = 1 Glucose is the primary energy source of an animal. RER = 0,7 fatty acids are the primary energy source. RER values in between suggest mixed fuel source.

All parameters have been normalized to lean body mass (LBM) measured by MRI. Asterisk (*) indicates statistical significance. Difference in means between *Ldlr$^{-/-}$ApoB*$^{100/100}$ and Chr4$^{\Delta70/\Delta70}$ groups and between light and dark hours was measured by using 2-way ANOVA with Tukey's multiple comparisons test, and the difference was considered statistically significant when adjusted $P < 0.05$.

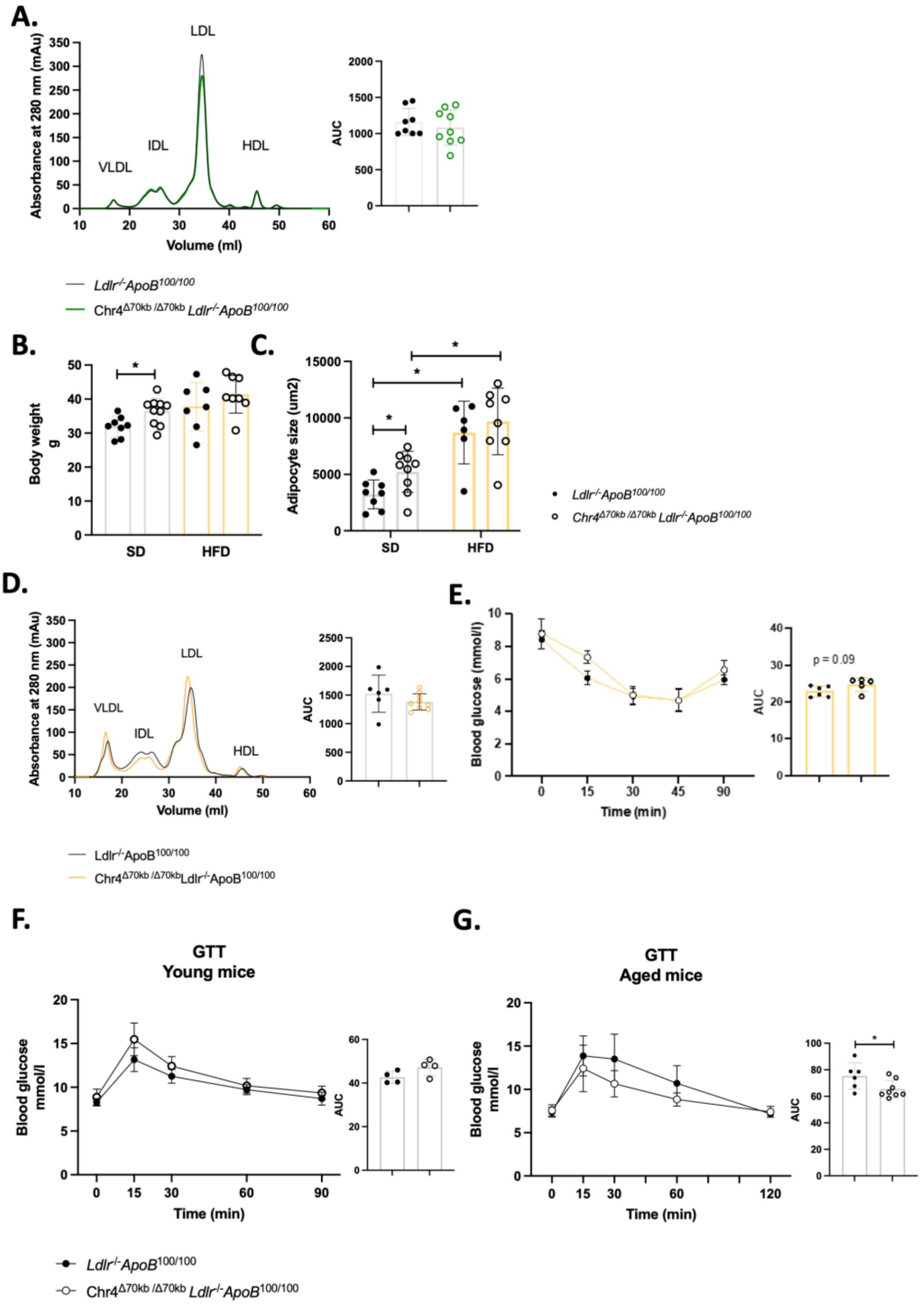

Supplemental Figure S2. Chr4$^{\Delta70/\Delta70}$ did not have effect on body weight gain, plasma lipoprotein profile, or insulin resistance after prolonged HFD in mice.

A) Plasma lipoprotein profile of aged Chr4$^{\Delta70/\Delta70}$ (n=9) and *Ldlr*$^{-/-}$*ApoB*$^{100/100}$ (n=8) mice on standard diet. **B)** Bodyweight and **C)** adipocyte size of aged Chr4$^{\Delta70/\Delta70}$ (n=10) and *Ldlr*$^{-/-}$*ApoB*$^{100/100}$ (n=8) mice on SD and HFD (n=8+6-7). **D)** Plasma lipoprotein profile of aged Chr4$^{\Delta70/\Delta70}$ (n=9) and *Ldlr*$^{-/-}$*ApoB*$^{100/100}$ (n=6) mice on HFD. **E)** Insulin tolerance test of aged of aged Chr4$^{\Delta70/\Delta70}$ (n= 6) and *Ldlr*$^{-/-}$*ApoB*$^{100/100}$ (n=6) mice on HFD. **F)** Glucose tolerance test of young Chr4$^{\Delta70/\Delta70}$ (n=4) and *Ldlr*$^{-/-}$*ApoB*$^{100/100}$ (n=4) mice on SD. **G)** Glucose tolerance test of aged Chr4$^{\Delta70/\Delta70}$ (n=8) and *Ldlr*$^{-/-}$*ApoB*$^{100/100}$ (n=6) mice on SD

Asterisk (*) indicates statistical significance. For graphs A, D, E, F and G, area under the curve (AUC) was first determined for each individual animal and difference in mean between groups was measured by using *t*-test. For graphs B-C, difference between SD and HFD groups was measured with ANOVA and Tukey's multiple comparisons test, and between SD with *t*-test. Differences were considered statistically significant when P or adjusted P < 0.05.
